# Clinical effect and safety of targeted therapy combined with chemotherapy in the treatment of patients with advanced colon cancer

**DOI:** 10.12669/pjms.39.4.7105

**Published:** 2023

**Authors:** Tao Zhang, Zhi Liu, Qian Lin

**Affiliations:** 1 Tao Zhang, Department of General Surgery, The No.2 Hospital of Baoding, Baoding 071051, Hebei, P.R. China; 2 Zhi Liu, Department of General Surgery, The No.2 Hospital of Baoding, Baoding 071051, Hebei, P.R. China; 3 Qian Lin, Department of Nursing, The No.2 Hospital of Baoding, Baoding 071051, Hebei, P.R. China

**Keywords:** Targeted therapy, Chemotherapy, Advanced colon cancer, Prognosis

## Abstract

**Objective:**

To evaluate the clinical effect and safety of immunotherapy combined with chemotherapy in patients with advanced colon cancer.

**Methods:**

This is a retrospective study. The subjects of this study were 120 patients with advanced colon cancer who were admitted to The No.2 Hospital of Baoding from November 30, 2019 to November 30, 2021. The enrolled patients were randomly divided into two groups, with 60 cases in each group. Patients in the control group were given F0LF0X4 regimen, while those in the study group were provided with Bevacizumab therapy on the basis of the method in the control group. All patients were evaluated after two cycles of treatment. The comparison of outcome measures included the curative effects, adverse drug reactions, improvement of quality-of-life scores and changes in tumor markers between the two groups.

**Results:**

The total effective rate of the study group was significantly better than that of the control group. There was no significant difference in the incidence of adverse drug reactions between the two groups. After treatment, the study group had a significantly higher rate of improved quality of life score, while the obviously lower rate of the aggravated score than those in the control group. The levels of CEA, CA19-9 and CA125 in the study group were significantly lower than those in the control group after treatment.

**Conclusion:**

Targeted therapy combined with chemotherapy is a safe and effective therapeutic option that has a definite curative effect in the treatment of patients with advanced colon cancer.

## INTRODUCTION

Colon cancer is a common digestive tract malignant tumor with a high incidence clinically, ranking fifth among malignant tumor diseases.^1^ It has no significant symptoms in the early stage, only including fecal occult blood, dyspepsia, etc. However, in case of progressing to the advanced stage, patients will show obvious symptoms such as abdominal distension, bloody stool, low fever, anemia and other adverse symptoms, which will threaten the safety-of-life of patients in serious cases.^2^ In clinical practice, surgery is considered to be the primary therapeutic option for the treatment of colon cancer.^3^ Nevertheless, due to the lack of specificity of symptoms in the early stage, some patients are often diagnosed at an advanced stage and already lost the opportunity of surgery. The main measures for the treatment of advanced colon cancer include chemotherapy, neoadjuvant therapy, radiotherapy and chemoradiotherapy. Significantly, the formulation of an effective chemotherapy regimen is of great importance in treating these patients.^4^

A prior randomized trial has documented that the FOLFIRI regimen can realize an excellent progression-free survival for patients with advanced colon cancer, which is better than 5-FU or other chemotherapeutics alone.^5^ While the serious side effects of chemotherapy are also a common problem that cannot be ignored since patients may show poorer tolerance to the therapy, and some patients even give up the treatment. Consequently, there may be poor curative effects and poor tolerance in patients, leading to poor improvement of patients’ symptoms. Significantly, molecular targeted drugs and chemotherapy drugs have been reported to have synergistic effects in the treatment of advanced tumors.^6^,^7^ At the same time, with the continuous improvement of symptoms, the tolerance of patients is higher. Bevacizumab is one of the molecular targeted agents that is commonly used clinically. Its mechanism of action is competitive binding with vascular endothelial growth factor (VEGF) receptor, inhibiting the migration and proliferation of endothelial cells, blocking angiogenesis and inhibiting tumor proliferation.^8^ At present, some reports show that bevacizumab combined with chemotherapy has certain benefits in the treatment of colon cancer.^9^,^10^ However, there is still a lack of high therapeutic research on the efficacy of bevacizumab combined with FOLFOX4 regimen in the treatment of advanced colon cancer. With respect to the above, the present study was performed to discuss the clinical effect and safety of targeted therapy combined with F0LF0X4 regimen in patients with advanced colon cancer.

## METHODS

In this retrospective study, clinical effect and safety of immunotherapy combined with chemotherapy in patients with advanced colon cancer. A total of 120 patients with advanced colon cancer who were admitted to The No.2 Hospital of Baoding from November 30, 2019 to November 30, 2021.The enrolled patients were randomly divided into two groups, with 60 cases in each group. This study has been approved by the medical ethics committee of The No.2 Hospital of Baoding (No.:HX2021040; date: November 30,2021), and written informed consent was obtained from all participants.

### Inclusion criteria:

- Patients with colon cancer diagnosed as stage III B ~ IV by pathological and imaging examination.^11^-^13^
- Patients with Karnofsky Performance Score (KPS) ≥80 points and estimated survival of >three months.
- Patients aged ≤75 years old.
- Patients with written informed consent, and patients with good treatment compliance and whose families were willing and able to cooperate to complete the study.

### Exclusion criteria:

- Patients with severe infectious diseases, immune diseases and serious dysfunction of important organs.
- Patients with malignant tumors of other systems.
- Patients with severe organic diseases or congenital diseases (such as valvular heart disease, severe pancreatitis, congenital heart disease, etc.).
- Patients with mental disorders or cognitive abnormalities who could not cooperate to complete the study.
- Patients who took relevant drugs (e.g., immunosuppressants, hormones, etc.) that might affect the study in the near future.
- Patients without contraindications to the drugs used in this study.

Before treatment, patients in both groups were given basic tests such as blood cell analysis, liver function and renal function, with the corresponding improvement of the abnormal indicators. Following pre-chemotherapy hydration one day before chemotherapy, patients in the control group were provided with an F0LF0X4 regimen, including Oxaliplatin (80mg/m^2^) in 5% glucose solution (250ml) for two hours of intravenous drip on Day one; formyltetrahydrofolate (200mg/m^2^) in 5% glucose solution (250ml) for two hours of intravenous drip on Day 1~2; and Fluorouracil Injection (400mg/m^2^) for 22 hours of intravenous injection through micro-pump continuously.^14^ The above therapeutic regimen was performed once every two weeks, and four weeks was a cycle. Patients were given antiemetic’s and drugs for increasing white cells during treatment. Simultaneously, patients in the study group were treated with an intravenous drip of Bevacizumab (5mg/kg) dissolved in 500ml normal saline on the basis of the treatment in the control group. Patients in this group were treated with two cycles (14 d/cycle).

### Outcome measures:

(1) *Evaluation of curative effect:* The curative effect was evaluated after two cycles of treatment,^15^ which was classified into the following types: complete remission (CR): Disappearance of the focus and normal recovery of the level of tumor markers for >four weeks; partial remission (PR): Decrease in the sum of length and diameter of the lesion by >30% and reduction in the level of tumor markers for >four weeks; no change (NC): No narrowing of the target focus, but with an increase in the sum of length and diameter within 20%, and no significant change in the level of tumor markers; and progressive disease (PD): Increase in the sum of baseline length and diameter of the lesion by >20%, and increase in the level of tumor markers. Overall response rate (RR) was calculated based on the formula of RR=CR+PR/CR+PR+NC+PD×100%. (2) Evaluation of adverse drug reactions.

The adverse reactions of the two groups of patients within one month after medication were recorded, including proteinuria, leucopenia, erythropenia, thrombocytopenia, gastrointestinal symptoms, etc. (3) *Quality of life score:* Eastern Cooperative Oncology Group (ECOG) score^16^ was used to observe the improvement of quality of life in the studied patients before and after treatment, including improved (score decrease ≥1 point’s), stable (no change in the score) and aggravated (score increase ≥1 point’s) quality of life scores. (4) Comparative analysis of tumor markers: Fasting blood was taken from each patient before and after treatment to detect carcinoembryonic antigen (CEA) (normal range < 5.0ng/ml), CA19-9 (normal range < 37U/ml) and CA125 (normal range < 35U/ml). The differences in these markers were further compared and analyzed between the two groups.

### Statistical analysis:

This study adopted SPSS 20.0 software for statistical analysis of all data. The measurement data were expressed in (*χ̅*±*S*). Inter-group comparison used two independent samples t-test, and intra-group analysis was realized using paired t-test. In addition, Chi-square (c^2^) test was used to compare the rates between groups. The difference was statistically significant when p<0.05.

## RESULTS

There were 37 males and 23 females in the study group, with an average of (68.27±5.38) years (63~73 years old); and the control group included 35 males and 25 females, with an average age of (67.73±6.52) years (61~75 years old). There was no significant difference in the general data between the study group and the control group, indicating the comparability between the groups (Table-I).

**Table-I:** Comparison of general data of the study group and the control group [(*χ̅*±*S*)., (n, %)].

Indexes	Study group	Control group	t/χ^2^	P
n	60	60		
Age (years)	68.27±5.38	67.73±6.52	0.49	0.62
Male (n %)	37 (62%)	35 (58%)	0.14	0.71
***Pathological type***				
Adenocarcinoma	33 (55%)	37 (62%)	0.55	0.46
Mucinous adenocarcinoma	19 (32%)	16 (27%)	0.36	0.55
Others	8 (13%)	7 (11%)	0.07	0.78
***Tumor site***			0.85	0.36
Left	32 (53%)	37 (62%)		
Right	28 (47%)	23 (38%)		
***Clinical stage***			0.20	0.66
IIIB	48 (80%)	46 (77%)		
IV	12 (20%)	14 (23%)		
***Degree of differentiation***				
High	22 (37%)	25 (42%)	0.31	0.58
Moderate	30 (50%)	29 (48%)	0.03	0.86
Low	8 (13%)	6 (10%)	0.32	0.57

P>0.05.

Shows the results of the comparison of curative effects between the two groups. The overall RR was 85% in the study group and 70% in the control group, which was significantly better in the former group than that in the latter group, with a statistically significant difference (p=0.03, Table-II).The incidence of adverse reactions was 30% in the study group and 22% in the control group. There was no significant difference in the incidence of adverse reactions between the two groups (p=0.30, Table-III).

**Table-II:** Comparison of the curative effect of the two groups of patients (n, %) .

Groups	CR	PR	NC	PD	RR
Study group (n=60)	17	34	6	3	51 (85%)
Control group (n=60)	11	31	12	6	42 (70%)
*c* ^2^	-	-	-	-	4.66
P	-	-	-	-	0.03

P<0.05.

***Notes:*** CR, remission; PR, partial remission; NC, no change; PD, progressive disease; RR,overall response rate.

**Table-III:** Comparison of adverse drug reactions between the two groups of patients (n, %).

Groups	Rash	Bone marrow suppression	Allergy	Fever	Abnormal liver function	Gastrointestinal reaction	Incidence
Study group (n=60)	2	5	3	2	3	3	18 (30%)
Control group (n=60)	0	4	3	1	1	4	13 (22%)
*c* ^2^	-	-	-	-	-	-	1.09
P	-	-	-	-	-	-	0.30

p>0.05.

After treatment, the study group had a significantly higher rate of improved quality of life score (p=0.03), while the obviously lower rate of the aggravated score (p=0.00) than those in the control group. Results in (Table-IV) suggested that the study group had obvious advantages in the improved quality of life score. Before treatment, no significant difference was detected in CEA, CA19-9 and CA125 levels between the two groups (p>0.05). While the levels of these indicators in the study group were significantly lower than those in the control group after treatment, with a statistically significant difference (p=0.00, Table-V).

**Table-IV:** Comparison of quality of life scores (determined by ECOG) between the two groups before and after treatment.

Groups	Improved*	Stable	Aggravated*
Study group (n=60)	48	9	3
Control group (n=60)	37	11	12
*c* ^2^	4.88	0.24	7.21
P	0.03	0.62	0.00

***Notes:***

* p<0.05.

**Table-V:** Comparison of tumor markers levels between the two groups before and after treatment (*χ̅*±*S*).

Indicators	Time of observation	Study group	Control group	t	p
CEA (ng/ml)	Before treatment	7.48±2.32	7.43±2.07	1.24	0.91
	After treatment*	3.27±0.93	5.92±1.07	14.48	0.00
CA19-9 (U/ml)	Before treatment	14.73±2.03	14.58±2.32	0.38	0.71
	After treatment*	4.32±1.06	7.88±2.01	12.14	0.00
CA125 (U/ml)	Before treatment	4.87±0.36	4.79±0.41	1.14	0.26
	After treatment*	1.86±0.07	3.05±0.23	13.84	0.00

***Notes:***

* p <0.05.

## DISCUSSION

In our study, patients with combination therapy in the study group had a higher RR rate and obviously improved quality of life scores after treatment. It can be speculated that for the treatment of advanced colon cancer, by making up for the deficiency of chemotherapy alone, Bevacizumab combined with chemotherapy can play a synergistic role and improve the anti-tumor effect. Colon cancer has been accepted to be a common digestive tract malignant tumor with a high incidence in middle-aged and elderly patients. Its pathological types mainly include adenocarcinoma, mucinous adenocarcinoma and undifferentiated carcinoma, with adenocarcinoma as the most common type.^17^ It is generally characterized by the lack of specific clinical symptoms in the early stage. Hence, some patients have developed into the advanced stage upon diagnosis, which means the loss of the surgery opportunity to achieve an effective therapeutic role.^18^ Medical treatment is the primary therapeutic option for patients with advanced colon cancer. Commonly, the use of chemotherapy can play a significant therapeutic effect, reduce the tumor size and further alleviate the clinical symptoms of patients.^19^ In addition, immunotherapy for colon cancer is also being carried out gradually.^20^

However, and notably, traditional chemotherapeutics are not favored by clinicians gradually owing to their poor therapeutic effect according to previous research.^21^ In recent decades, combined chemotherapy involving Oxaliplatin is extensively applied in clinical treatment, with the achievement of obvious therapeutic results.^22^ Oxaliplatin is a novel platinum derivative, which has significantly less toxicity to kidney and bone marrow than carboplatin and cisplatin and can effectively inhibit tumor growth. In combination therapy, besides improving the effect of other chemotherapeutics, it can also alleviate the adverse reactions caused by chemotherapy. It has been reported to have established anti-tumor activity in advanced or metastatic malignant tumor diseases.^23^

Furthermore, five-fluorouracil has higher activity and higher accuracy for tumor cells. Besides, it has less toxicity and will not cause adverse effects on the human body at a normal dose. Therefore, the combination of Oxaliplatin and five-fluorouracil may exhibit some advantage in treatment.^24^ However, additional researchers also considered a relatively unsatisfactory effect of chemotherapy alone.^25^ In terms of the major causes, tumor cells may spread widely in patients with advanced colon cancer, leading to a higher risk of complications, poor overall resistance of the body and compromised immunity in these patients. Consequently, these patients can’t bear the adverse reactions caused by chemotherapy.

Bevacizumab is a targeted drug that can be applicable for the treatment of various tumors. It can play a role by targeting and inhibiting the biological activity of VEGF. The use of Bevacizumab can reduce tumor angiogenesis and inhibit the progress of metastasis, which has been approved for the treatment of metastatic colorectal cancer, non-small cell lung cancer, etc.^26^ Concerning its mechanism of action, Bevacizumab can bind to VEGF released by tumor, prevent VEGF from binding to specific sites on vascular endothelial cells (VEGF receptors) to inhibit tumor angiogenesis, so as to disrupt the supply of nutrients to tumor cells and achieve the purpose of inhibiting tumor growth.^27^ The application of Bevacizumab can induce rapid tumor regression, restore the structure of surviving tumor vasculature simultaneously, and reduce intratumoral pressure, thereby facilitating the delivery of chemotherapeutics to exert anti-tumor effect.^28^

It may be related to the fact that Bevacizumab can reverse the immunosuppression caused by chemotherapy and enhance the resistance of the body,^29^ resulting in reduced adverse reactions to some extent. Sakaguchi et al.^30^ pointed out that oxaliplatin-based chemotherapy plus bevacizumab and laparoscopic resection could be very effective for locally advanced colon cancer. On the basis of surgical treatment, Yonemitsu et al.^31^ used bevacizumab combined with chemotherapy to effectively treat a case of juvenile colon cancer with peritoneal dissemination. According to Mikami et al.^32^, chemotherapy with bevacizumab/FOLFOX4 plays a role in the management of advanced/unresectable colon cancer.

Furthermore, there was no significant difference in adverse drug reactions between the study group and the control group after treatment. It is suggested that targeted therapy of Bevacizumab combined with chemotherapy exhibit certain safety without aggravation in adverse reactions, which is basically consistent with the report of Ranieri et al.^33^ In addition, the level of tumor markers in the study group was significantly lower than that in the control group after treatment. Accordingly, Bevacizumab combined with oxaliplatin and fluorouracil can play a synergistic effect by compensating for the deficiency of chemotherapy alone and enhance the anti-tumor effect in the treatment of advanced colon cancer. Our results provide a reference for the application of bevacizumab combined with chemotherapy in the treatment of advanced colon cancer.

### Limitation of the study:

However, the present study still has some limitations that deserve to be emphasized, such as the small sample size and short follow-up period. Our future research will be continued based on the expanded sample size and prolonged duration of follow-up. Through expanded investigation, it is expected to further elaborate on the impact of different treatment schemes on the long-term effect and survival of patients, so as to evaluate the benefits of the proposed therapeutic schedule to patients more comprehensively.

## CONCLUSION

In conclusion, findings in our study suggest that targeted therapy combined with chemotherapy is a safe and effective treatment with obvious curative effects in the treatment of patients with advanced colon cancer. It can contribute to the significant improvement of quality of life and reduced levels of tumor markers, without an obvious increase in adverse reactions.

### Authors’ Contributions:

**TZ, ZL and QL:** Carried out the studies, participated in collecting data, and drafted the manuscript, and are responsible and accountable for the accuracy or integrity of the work. **TZ and QL:** Performed the statistical analysis and participated in its design.

All authors read and approved the final manuscript.
